# The Promotoer, a brain-computer interface-assisted intervention to promote upper limb functional motor recovery after stroke: a statistical analysis plan for a randomized controlled trial

**DOI:** 10.1186/s13063-023-07773-4

**Published:** 2023-11-16

**Authors:** Marta Cipriani, Floriana Pichiorri, Emma Colamarino, Jlenia Toppi, Federica Tamburella, Matteo Lorusso, Alessandra Bigioni, Giovanni Morone, Francesco Tomaiuolo, Filippo Santoro, Daniele Cordella, Marco Molinari, Febo Cincotti, Donatella Mattia, Maria Puopolo

**Affiliations:** 1 Department of Neuroscience, Istituto Superiore di Sanità, Rome, Italy; 2 Department of Statistical Sciences, Sapienza University of Rome, Rome, Italy; 3 Fondazione Santa Lucia, IRCCS, Rome, Italy; 4 Department of Computer, Control, and Management Engineering “Antonio Ruberti”, Sapienza University of Rome, Rome, Italy; 5 Department of Clinical and Experimental Medicine, University of Messina, Messina, Italy; 6 Research Coordination and Support Service, Istituto Superiore di Sanità, Rome, Italy; 7 IT Service, Istituto Superiore di Sanità, Rome, Italy

**Keywords:** EEG-based brain-computer interface, Stroke, Hand functional motor recovery, Neurorehabilitation, Randomized controlled trial, Statistical analysis plan

## Abstract

**Background:**

Electroencephalography (EEG)-based brain-computer interfaces (BCIs) allow to modulate the sensorimotor rhythms and are emerging technologies for promoting post-stroke motor function recovery. The Promotoer study aims to assess the short and long-term efficacy of the Promotoer system, an EEG-based BCI assisting motor imagery (MI) practice, in enhancing post-stroke functional hand motor recovery. This paper details the statistical analysis plan of the Promotoer study.

**Methods:**

The Promotoer study is a randomized, controlled, assessor-blinded, single-centre, superiority trial, with two parallel groups and a 1:1 allocation ratio. Subacute stroke patients are randomized to EEG-based BCI-assisted MI training or to MI training alone (i.e. no BCI). An internal pilot study for sample size re-assessment is planned. The primary outcome is the effectiveness of the Upper Extremity Fugl-Meyer Assessment (UE-FMA) score. Secondary outcomes include clinical, functional, and user experience scores assessed at the end of intervention and at follow-up. Neurophysiological assessments are also planned. Effectiveness formulas have been specified, and intention-to-treat and per-protocol populations have been defined. Statistical methods for comparisons of groups and for development of a predictive score of significant improvement are described. Explorative subgroup analyses and methodology to handle missing data are considered.

**Discussion:**

The Promotoer study will provide robust evidence for the short/long-term efficacy of the Promotoer system in subacute stroke patients undergoing a rehabilitation program. Moreover, the development of a predictive score of response will allow transferring of the Promotoer system to optimal clinical practice. By carefully describing the statistical principles and procedures, the statistical analysis plan provides transparency in the analysis of data.

**Trial registration:**

ClinicalTrials.gov NCT04353297. Registered on April 15, 2020.

## Introduction

Stroke is a leading cause of long-term disability with substantial social costs [1–3]. After experiencing a stroke, individuals often suffer from upper limb motor dysfunction, which leads to long-term disability [4]. The extent of upper extremity function is a crucial predictor of the patient’s ability to return to work [5]. Despite extensive therapy, variations in upper limb recovery patterns continue to significantly impact rehabilitation outcomes [6], and cost-effective post-stroke rehabilitation programs for the upper limbs are critically needed.

Electroencephalography (EEG)-based brain-computer interface (BCI) is an emerging technology that enables the direct translation of brain activity into physical motion [7] and is considered as a promising tool to promote functional motor recovery of the upper limbs after a stroke [8].

In a pilot study, an EEG-based BCI-assisted MI training (BCI-MI) administered using a BCI system fully compliant with rehabilitation requirements—the Promotoer system—proved to be useful in enhancing post-stroke functional hand motor recovery [9]. However, robust evidence for short- and long-term efficacy and availability of quantifiable indices (predictors) of patient response is needed for BCI-MI application to clinical practice. To this end, a randomized controlled study, named the Promotoer study, was conceived in subacute stroke participants.

The primary objective of the “Promotoer” trial is to determine whether the BCI-MI administered using a BCI system fully compatible with a clinical setting (the Promotoer) is superior to a non-BCI assisted MI training (Control-MI) in improving hand motor function outcomes in sub-acute stroke patients admitted to the hospital for their standard rehabilitation care. The secondary objectives of this study are as follows: (i) to assess the long-term efficacy of the BCI-based intervention on hand motor function outcome (6 months follow-up) and (ii) to determine if the clinical improvement is accompanied by a long-lasting neuroplasticity change. Moreover, the study aims to identify biomarkers and potential predictors of patient responses to the BCI-Promotoer training.

The protocol of the study, providing detailed information on background, study design, experimental intervention, and clinical procedures, was published previously [10]. The study was approved by the Independent Ethical Committee of the Fondazione Santa Lucia (FSL) IRCCS, Rome, Italy (CE/PROG.755), and is being conducted in accordance with the Helsinki Declaration and current national regulation on clinical trials, with high priority given to patient safety. The study has been registered at www.clinicaltrials.gov (NCT04353297), and its status is recruiting.

In this article, we report the details of the statistical analysis plan (SAP) of the Promotoer study (SAP Version 1.0 July 13, 2023), based on information contained in the PROMOTOER Clinical Protocol Version 1.0 [10], by carefully describing statistical principles and procedures for the analysis of data and presentation of the study results. The document follows the guidelines for the content of statistical analysis plans in clinical trials [11] and is in full compliance with the International Conference on Harmonisation ICH E9 guidance [12].

## Study methods

### Trial design

The Promotoer trial is designed as a randomized, controlled, assessor-blinded, single-centre, superiority trial with two parallel groups with a 1:1 allocation ratio. The two intervention groups are the BCI-assisted MI training (experimental group, BCI-MI) and the MI training not supported by BCI (control group, Control-MI). Both interventions will comprise a total of twelve 45-min sessions with a delivery of three times per week. Training will be completed in 4–6 weeks. The study interventions are conceived as add-on regimen to standard rehabilitation care (details are given in the published study protocol [10]).

Participant recruitment, intervention delivery, and data collection will take place at Fondazione Santa Lucia IRCCS, Rome, Italy.

### Randomization

Participants are assigned to either the experimental group (BCI-MI) or the control group (Control-MI) in a 1:1 ratio through a randomized allocation process. This randomization process has been stratified by the side of the stroke lesion (left or right hemisphere) and the baseline score of UE-FMA/60, with 60 as the maximum score (< 19, severe; from 20 to 47, moderate) [13].

To carry out the randomization, a list has been generated using permuted blocks of varying sizes through the Ralloc procedure of the Statistical software STATA (https://www.stata.com/). The randomization list was centrally generated by the trial statistician at Istituto Superiore di Sanità (ISS, Rome Italy) and then securely managed. To maintain the integrity of the randomization process, numbered, opaque, sealed envelopes were prepared to replicate the randomization list for each specific stratum. These envelopes were delivered to the clinician responsible for participant randomization at Fondazione Santa Lucia IRCCS.

When a patient becomes eligible for randomization, the clinician in charge submits a randomization form containing the relevant stratification data and the date of the request. Then, the clinician responsible for participant randomization at Fondazione Santa Lucia IRCCS assigns to the patient the envelope with the number corresponding to the progressive order of randomization of the patient in his/her stratum.

### Sample size

The results of the pilot study [8] demonstrated that the BCI-MI group had significantly higher effectiveness in terms of FMA scores compared to the control group (mean ± SD: 44 ± 34.7 vs. 19.8 ± 19.8). This effect size was considered clinically relevant and used for sample size calculation, which was carried out with a one-sided alpha level of 5% for the test of superiority, a statistical power of 80%, and a *t-test* for independent groups. The calculation revealed that each group should consist of 18 participants. To account for potential loss to follow-up, a dropout rate of 25% was considered, leading to a total of 48 patients required for the study. The sample size calculation was conducted using the *power twomeans* module in the Stata/MP 17.0 statistical software.

### Framework

The primary objective of the trial is to evaluate the superiority of BCI-MI training (BCI-MI) against non-BCI assisted MI training (Control-MI) in improving hand motor function outcomes in sub-acute stroke patients admitted to the hospital for their standard rehabilitation care. Thus, the research hypothesis is settled in a superiority framework.

### Statistical interim analysis and stopping guidance

To check the congruity of nuisance parameters used in sample size calculation, an internal pilot study (IPS) for sample-size recalculation was planned on data of primary outcome in the first 20 participants. Specifically, estimates of standard deviations of the effectiveness of the FMA score at the end of intervention training (T1) in the intervention group will be obtained from masked IPS data and used in the sample size procedure specified in the “Sample size” section, considering other assumptions and data as unchanged. Only upward adjustments of the initially planned sample size will be allowed, according to restricted IPS [14]. R code used for masked calculation of standard deviations is reported in the Appendix.

No interim efficacy analysis is planned. Pockock method for control of family-wise type I might be implemented by using the software RPACK (Confirmatory Adaptive Clinical Trial Design and Analysis, R package), if an interim analysis is required due to changes in the recruitment conditions.

### Timing of final analysis

The final analyses will be performed after completion of the last visit of the last enrolled patient. The time schedule of trial assessments is reported in Table 1. Data for analyses will be extracted from the electronic case report form (eCRF) used for data entry and monitoring during the clinical trial.

**Table 1:** Time schedule of assessments of baseline and outcomes data

Data collection instrument	Enrolment	Randomization	Evaluation T0	Training session												Evaluation			
				**1**	**2**	**3**	**4**	**5**	**6**	**7**	**8**	**9**	**10**	**11**	**12**	**T1**	**T2**	**T3**	**T4**
Demographic and clinical data	●																		
Token test	●																		
FMA/60	●																		
Eligibility criteria	●																		
Informed consent	●																		
Randomization		●																	
Edinburgh handedness inventory			●																
EEG			●													●	●	●	●
MEP			●																
Neuropsychological assessments			●																
MRI			●															●	●
FMA/66			●													●	●	●	●
NIHSS			●													●	●	●	●
ARAT			●													●	●	●	●
MAS			●													●	●	●	●
MMT			●													●	●	●	●
NRS pain			●													●	●	●	●
Training session, run				●	●	●	●	●	●	●	●	●	●	●	●				
QCM					●	●	●	●	●	●	●	●	●	●	●				
VAS Mood					●	●	●	●	●	●	●	●	●	●	●				
NASA TLX					●										●				
VAS Satisfaction					●	●	●	●	●	●	●	●	●	●	●				
QUEST															●				
SUS															●				
Status			●	●	●	●	●	●	●	●	●	●	●	●	●	●	●	●	●
Rehabilitation monitoring																●	●	●	●

*FMA* Fugl-Meyer Assessment, *EEG* Electroencephalography, *MEP* Motor evoked potentials, *MRI* Magnetic resonance imaging, *NIHSS* National Institute of Health Stroke Scale, *ARAT* Action Research Arm Test, *MAS* Modified Ashworth Scale, *MMT* Manual Muscle Test, *NRS* Numeric rating scale, *QCM* Questionnaire on Current Motivation, *VAS Mood* Visual Analogue Scale of Mood, *NASA TLX* NASA Task Load Index, *VAS Satisfaction* Visual Analogue Scale of Satisfaction, *QUEST* Quebec User Evaluation of Satisfaction with assistive Technology 2.0, *SUS* System Usability Score, T0 Evaluation at randomization, before intervention, T1 at end of intervention training (within 48 h); *T2*, at 1 month from end of training; *T3*, at 3 months from end of training; *T4*, at 6 months from end of training

Blinding will be removed in two steps: analysis will be performed upon the study’s completion and the database’s freezing, using group assignments as group A and group B. When all analyses have been performed and the final report drafted, BCI-MI and Control-MI will be un-blinded.

### Statistical principles

#### Confidence intervals and P values

The primary hypothesis testing will be performed at a one-sided alpha of 0.05. The significance level used for the primary outcome is consistent with that used in the sample size calculation. Hypothesis testing on any of the secondary outcomes will be performed at a two-sided alpha of 0.05. Treatment effects will be reported through mean differences for continuous variables and odds ratio for binary variables, with two-sided 95% confidence intervals.

Adjustment for multiplicity will be considered in post-hoc comparisons of secondary analyses, specifically in comparisons among groups in each evaluation time (T1, T2, T3, and T4). Bonferroni’s correction criteria will be applied, and exact *P*-values will be reported.

#### Adherence and protocol deviations

Adherence to study interventions (BCI-MI or Control-MI) is fixed as a minimum of 9 training sessions (out of 12) delivered within 6 weeks. Moreover, protocol deviations include loss at follow-up (withdrawal/drop-out/death for any cause) and/or missed assessment for primary outcome at T1. The number of patients with adherence to the study intervention and the number of patients showing deviations from the planned study intervention will be reported for each intervention group. Deviations will be described for each patient by intervention group.

Major protocol deviations are missing adherence to study interventions.

#### Analysis populations

The as-randomized (AR) population includes all randomized patients, regardless of subsequent withdrawal from training sessions or deviation from the study protocol.

The intention-to-treat (ITT) population is defined as all randomized participants who will perform (minimum) one training session (both for BCI-MI training and Control-MI training intervention groups).

The per-protocol (PP) population includes those randomized participants without major protocol deviations, namely patients with adherence to the study intervention.

The user experience (UE) population includes all patients randomized and assessed for user experience.

### Trial population

#### Screening and eligibility

Screening for eligibility and enrolment in the study are detailed in the study protocol [10]. Briefly, eligible candidates are patients of any gender, aged 18 to 80 years, with a first-ever unilateral stroke confirmed by magnetic resonance imaging (MRI), that occurred from 1 to 6 months before admission to the Fondazione Santa Lucia IRCCS site for rehabilitation care. The eligible patients must show hemiplegia/hemiparesis of the upper extremity and a UE-FMA/60 score (out of a maximum 60 score) lower or equal to 47 [participants with severe symptoms (scores from 0 to 19) and moderate symptoms (score from 20 to 47) will be included]. The main exclusion criteria include dementia, severe neglect, or severe aphasia, severe spasticity (Modified Ashworth Scale > 4 at shoulder/elbow/wrist), a UE-FMA/60 score greater than > 47, a Token Test (TT) score lower or equal to 29, and concomitant neurological disorders. All eligibility criteria must be met by the time of the screening visit.

#### Recruitment, follow-up, and withdrawal

Patients who meet the eligibility criteria and have provided informed consent will be randomized and will undergo the planned evaluations at T0 (see Table 1). Any participant who enrolled in the study can withdraw from the study at any time, for any reason, without prejudice or consequence. The status of participants will be monitored during each scheduled training session and evaluation visit. In cases where a patient misses a planned evaluation visit, a phone call will be made to check their status.

**Fig. 1:**
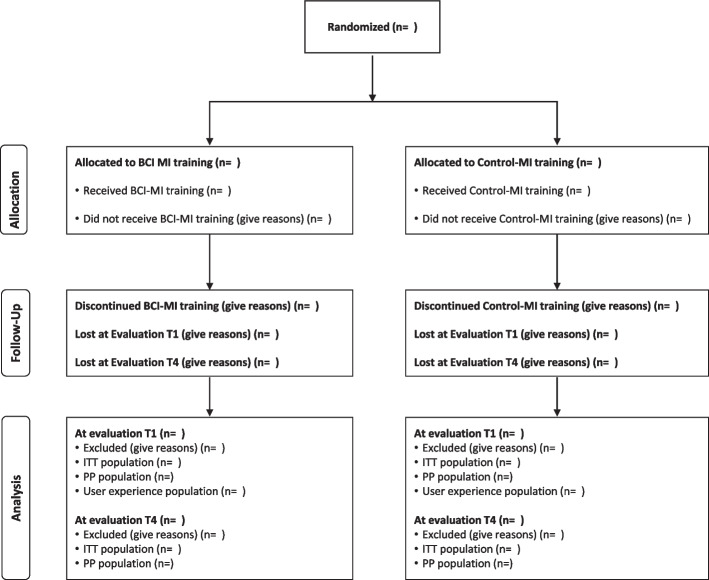
Promotoer CONSORT flow diagram (CONSORT 2010)

A CONSORT flow diagram specifically designed for the Promotoer study (Fig. 1) will be used to summarize the numbers of patients who were randomized, received their allocated intervention, withdrew, or were lost to follow-up and were included in population analysis at T1 and T4 evaluation visits.

The timing of withdrawals and loss to follow-up, whenever possible, will be presented as time-to-event data and analysed using Kaplan–Meier curves, stratified by intervention group. The Log-rank test will be employed to assess any potential differences between the two curves. Furthermore, the reasons for the loss of participants during the study will be described for each intervention group.

The data collected from participants who have withdrawn from the study will be included in the analysis sets, unless the participants expressly withdraw their consent for the use of data collected until that time point.

#### Baseline patient characteristics

Following the time schedule shown in Table 1, patient characteristics in the two randomized groups will include:

- Demographical data (sex, age at enrolment)
- Clinical data and functional assessments collected at the screening visit for eligibility (type and side of lesion, TT score, FMA/60 upper limb section score)
- Functional assessments completed at evaluation time T0 after the signature of the informed consent, (MEP, TMT, TAP, Edinburgh handedness inventory score, UE-FMA 66, NIHSS, ARAT, MMT for shoulder/elbow/wrist, NRS for pain for affected upper limb, and the MAS for spasticity score for shoulder/elbow/wrist)

All continuous variables will be summarized by descriptive statistics of *n*, mean, standard deviation, minimum, maximum, median, and interquartile range. Shapiro Wilk’s test will be used to assess the normal distribution of variables. Categorical data will be summarized by frequencies and percentages. Test of statistical significance will not be undertaken for baseline characteristics; rather, the clinical importance of any imbalance will be noted.

A table detailing baseline characteristics will be compiled for the ITT population (as outlined in the “Analysis populations” section). Additionally, separate tables of baseline characteristics will be generated for the AR and PP populations. These tables will be useful to assess the success of randomization by examining the balance of prognostic factors among the different intervention groups and to verify whether any attrition during the study has introduced a selection bias or has disrupted the balance that was initially achieved through randomization.

### Analysis

#### Outcome definitions

The primary outcome is the “effectiveness” of the UE-FMA score at T1. The UE-FMA is widely recommended for the evaluation of sensorimotor impairments of the upper extremity in stroke rehabilitation research [9, 15, 16]. It ranges from 0 to 66 (best score). The effectiveness of the UE-FMA score is defined as the proportion of potential improvement that could be achieved after the intervention and is calculated as score at T1 minus score at T0, divided by the maximum score (66) minus score at T0, multiplied by 100 (as specified in [9]) [formula: 100 × (UE-FMA at T1 − UE-FMA at T0)/(66 − UE-FMA at T0)]. Thus, if a patient achieves the highest possible score after the intervention, the effectiveness is 100%. This approach allows to normalize the data, taking into account baseline data. The secondary outcomes are presented in Table 2. Electroencephalogram (EEG) and MRI data will be considered in a separate document.

**Table 2:** Secondary outcomes

Outcome	Description
**Clinical and functional outcomes**	
Effectiveness of Fugl-Meyer Assessment of Upper Extremity (UE-FMA) score at evaluation times T2, T3, and T4	Analogously to the primary outcome, the effectiveness [9, 17] of the UE-FMA score is the proportion of potential improvement that could be achieved after the intervention in the UE-FMA score: $100\times \frac{UEFMA at {T}_{i}-UEFMA at {T}_{0}}{66-UEFMA at {T}_{0}}$ where $i=2, 3, 4.$
Minimal clinically important difference (MCID) at evaluation times T1, T2, T3, and T4	MCID is the improvement of at least 7 points in UE-FMA, with respect to baseline [18]
MAS for spasticity score for shoulder/elbow/wrist stroke at evaluation times T1, T2, T3, and T4	MAS score is used to assess muscle tone and spasticity in individuals with neurological conditions, such as cerebral palsy or stroke [19]. It evaluates resistance to passive movement in specific muscle groups. It ranges from 0 to 4
Numeric rating scale (NRS) for pain at evaluation times T1, T2, T3, and T4	NRS score is commonly used to assess the intensity of pain experienced by a person. It is a self-report score and ranges from 0 (“no pain”) to 10 (“the worst pain imaginable”)
Effectiveness of Action Research Arm Test (ARAT) score for upper limb function at evaluation times T1, T2, T3, and T4	ARAT score [20] is used for assessing upper limb function. It ranges from 0 to 57. The effectiveness [17] of ARAT score is the proportion of potential improvement that could be achieved after the intervention: $100\times \frac{ARAT at {T}_{i}-ARAT at {T}_{0}}{57-ARAT at {T}_{0}}$ where $i=1, 2, 3, 4.$
Effectiveness of the National Institute of Health Stroke Scale (NIHSS) score at evaluation times T1, T2, T3, and T4	NIHSS [21] is used to evaluate the severity of a stroke and to measure the level of neurological impairment in stroke patients. It ranges from 0 (no neurological deficit) to 42 (severe neurological deficit). Effectiveness [17] of NIHSS score is the proportion of potential improvement in NIHSS that could be achieved after the intervention: $100\times \frac{NIHSS at {T}_{i}-NIHSS at {T}_{0}}{42-NIHSS at {T}_{0}}$ where $i=1, 2, 3, 4.$
Effectiveness of Manual Muscle Test (MMT) score for shoulder/elbow/wrist (flexor/extensor muscles), at evaluation times T1, T2, T3, and T4	MMT [22] is used to evaluate muscle function and monitor changes in muscle strength over time. The maximum possible score for shoulder/elbow/wrist (flexor/extensor muscles) is 25 Effectiveness [17] of MMT score is the proportion of potential improvement that could be achieved respect to the baseline: $100\times \frac{MMT at {T}_{i}-MMT at {T}_{0}}{25-MMT at {T}_{0}}$ where $i=1, 2, 3, 4.$
**User experience outcomes**	
Questionnaire on Current Motivation (QCM, 4 items) score, at training sessions 2 to 12	The QCM [23] score is used to evaluate motivation and adherence to technology
Workload NASA-TLX Total score at training sessions 2 to 12	The Workload NASA-TLX Total score [24] is used to evaluate mental workload fatigue during training with technology
System Usability Score (SUS) at training session 12	The SUS [25] is used to evaluate the usability of technology
Quebec User Evaluation of Satisfaction with assistive Technology 2.0 (QUEST 2.0), at training session 12	The QUEST 2.0 score [26] is used to evaluate satisfaction with technology
Visual Analogue Scale for Mood (VAS Mood) score at training sessions 1 to 12	VAS for Mood is a self-assessment tool used to measure a person’s emotional state or mood
Visual Analogue Scale for Satisfaction (VAS Satisfaction) score at training sessions 1 to 12	VAS for Satisfaction is a self-assessment tool used to measure an individual’s level of satisfaction with a particular experience, product, service, or intervention

#### Analysis methods for primary and secondary outcomes

The primary analysis will be carried out on the primary outcome in the ITT population—namely effectiveness of the UE-FMA at T1. In the secondary analysis, the analysis of the primary outcome will be carried out in the PP population. The secondary outcomes will be compared between groups both in the ITT and PP populations.

For continuous variables (effectiveness of UE-FMA score, of ARAT score, of NIHSS score, of MMT score, NRS, QCM, Workload NASA-TLX, SUS, QUEST, VAS Mood, VAS Satisfaction) descriptive statistics (mean and standard deviation, median and interquartile range) will be reported by intervention group for each evaluation time/training session. *t-test* for independent groups will be used for comparisons of the BCI-MI group vs Control-MI group in each evaluation time/training session. Shapiro–Wilk’s test will be used to assess the normal distribution, and the Mann–Whitney test will be used for comparison if the normality assumption is violated.

To determine whether and to what extent the long-term efficacy of the BCI intervention on the UE-FMA score can be maintained after the end of the intervention, we will use a repeated measures analysis of variance (ANOVA). ANOVA will treat the group as a between-subject factor and time as a within-subject factor, assessing the effectiveness of the UE-FMA scores at T1, T2, T3, and T4.

All categorical variables will be summarized (by number and frequency) in each intervention group and compared between groups by Fisher’s test, at each evaluation time (T2, T3, T4).

To identify clinical and/or neurophysiological determinants of MCID (Table 2) in the participant response to an intervention at T1, a forward-stepwise binary logistic regression will be performed including the intervention group, demographical, clinical, and neurophysiological parameters. Odds ratios and 95% confidence intervals will be calculated. To reduce the dimensional space of available variables, variable selection algorithms (e.g. least absolute shrinkage and selection operator—LASSO regression) will be used to select the most informative features to be included in the predictive model. A probability score will be defined to assess the likelihood of good recovery based on obtained determinants of response. A further logistic regression analysis will be performed including only the BCI-MI group. The same strategies of analysis will be used for the evaluation of MCID at all subsequent evaluation times (T2, T3, T4).

Statistical analysis of the MAS score will be detailed for shoulder, elbow, and wrist. Descriptive statistics on frequency distributions will be reported for each time point (T1, T2, T3 and T4) and compared between intervention groups by chi-square test. Moreover, a change in the score between T0 and T1, and changes along the follow-up (e.g. T2 vs T0; T3 vs T0, T3 vs T0, and T4 vs T0) will be computed and defined as improved/stable/deteriorated. Such changes will be compared in the intervention groups using a chi-square test or Fisher’s exact test.

Scores obtained in user experience assessments for QCM (separately for Mastery confidence, Incompetence/Fear-to Fall, Challenge and Interest), Workload NASA-TLX, SUS, QUEST, VAS Mood, and VAS Satisfaction, will be described for each experimental group and comparisons will be carried out using the methodologies adopted for other continuous variables. Analyses will be carried out by training sessions.

#### Subgroup analyses

To investigate the possible effect of the number/amount of training sessions received, comparisons among sub-groups of BCI-MI participants defined according to the number of completed training sessions (< 9 vs >  = 9) will be carried out on primary and secondary outcomes using the same statistical methodologies adopted in the primary and secondary analyses.

Moreover, to assess the robustness of the effects of BCI-MI intervention, explorative subgroup analyses [27] will be carried out on the primary outcome in subgroups of participants identified by the following: baseline score of UE-FMA/60 with 60 as maximum score (< 19, severe; from 20 to 47, moderate); side of stroke lesion (left/right hemisphere); age at enrolment (< = 50 years vs > 50 years); time from stroke to admission to Fondazione Santa Lucia IRCCS site for rehabilitation care (< = 3 months vs > 3 months). Estimates and confidence intervals will be obtained for each subgroup and will be presented through forest plots. Moreover, for each subgroup variable, statistical tests for interaction [12, 28] will be carried out using ANOVA including the intervention group (BCI-MI vs Control-MI) and its interaction with the subgroup covariate. Exact *P*-values of the test of interaction will be reported to allow detection of signals for further inspection.

#### Missing data

The primary analysis will be performed on all available cases. During the conduction of the trial, data collection will be monitored to minimize missing data for the primary outcome. Moreover, we have planned sensitivity analyses for addressing missing data [29] pertaining to the primary outcome. These analyses will allow to evaluate the robustness of study findings.

The following data will be obtained separately for each intervention group:

- Proportion of participants with missing data for the primary outcome
- Comparison of baseline characteristics between participants with available data and participants with missing data for the primary outcome

If no important difference in baseline characteristics is observed between participants with available data and participants with missing data for the primary outcome, comparisons of intervention groups for the primary outcome will be carried out using multiple imputation techniques for missing data [30]. The estimation model will include variables used in the stratification of patients at randomization (baseline score of UE-FMA/60 and side of stroke lesion), age at enrolment and gender; 20 imputations will be obtained.

In case of a relevant amount of missing data (more than 25% of dropouts), a revision of the SAP will be necessary.

#### Safety analysis

BCI-MI and Control-MI interventions are not expected to yield adverse effects, as they are non-invasive procedures without the administration of drugs. Moreover, both interventions will be administered by trained professionals, including physiotherapists and neurophysiology technicians with expertise in EEG recordings, ensuring the safety and well-being of the participants. Previous experience with BCI training delivery in subacute stroke participants is encouraging since training was well-tolerated by the participants and no dropouts were reported.

#### Statistical software

Analyses will be carried out by the STATA 17 and R software (version 4.3.0).

#### Data collection and management

The study data are collected and managed using REDCap electronic data capture tools [31, 32] hosted at Istituto Superiore di Sanità (Rome, Italy). An ad hoc REDCap template was developed in line with the case report form (CRF) of the Promotoer study. This includes two sections: (1) the “Baseline and Randomization Section” including demographical and clinical baseline data, randomization data, and training session data, and (2) the “Outcomes Section” including baseline neurophysiological assessments and clinical and functional outcomes data at the planned evaluation times. Section 1 data are filled in by unblinded personnel, and Sect. 2 data are filled in by outcome assessors blinded to assigned intervention.

Automated export procedures for seamless data downloads to STATA and R statistical packages are available.

## Discussion

The Promotoer study is a randomized controlled study conceived to produce robust evidence for short and long-term efficacy of the EEG-based BCI-assisted MI training (the Promotoer system) and identify accurate indices (predictors) of response to this intervention in subacute stroke patients undergoing rehabilitation. Rigorous methodologies have been adopted in the study design, such as clear definitions of the experimental and control interventions, randomization of patients to the intervention groups, and blinded outcome assessment. The effect size considered in the sample size calculation was derived from the findings of the pilot study [8] and was considered clinically relevant and useful to support the translation of the Promotoer system to the clinical practice in neurorehabilitation. The trial has been registered on the ClinicalTrials.gov website (NCT04353297), and a detailed study protocol [10] has been developed according to SPIRIT guidance [33]. This paper describes the SAP of the Promotoer study, which was developed following the Guidelines for the Content of Statistical Analysis Plans in Clinical Trials [11]. Study methods, statistical principles, trial populations, and analysis issues have been detailed in line with the objectives of the study and the study design, to support transparency and reproducibility of data analysis.

## Conclusion

There is a strong need to improve the quality of conduct and reporting of research studies in rehabilitation [34], and a Randomized Controlled Trial Rehabilitation Checklists (RCTRACK) project is ongoing aiming at producing a specific reporting guideline for randomized controlled trials (RCTs) in rehabilitation [35].

Registration of the study, production of the study protocol and statistical analysis plan, and adherence to reporting guidelines are well-known, good clinical research practices [36, 37]. However, it is the responsibility of researchers to actively integrate them into their everyday research practices. The availability of SAP ensures the integrity and credibility of study results, guarding against selective reporting of outcomes and analyses, facilitating reproducible research, and increasing trust in science. Producing SAP has a pivotal role in upholding the quality of rehabilitation research, enhancing the reliability of findings, and ultimately contributing to evidence-based decision-making.

### Trial status

Recruitment status: ongoing recruitment.

Recruitment start date: 24 November 2020 (first randomization on 27 November 2020).

## Data Availability

Not applicable.

## Appendix

Script for blinded estimation of the standard deviation of the effectiveness of UE-FMA/66 at T1 by the intervention group in R language.

# Load the dataset (3 columns: record id, endpoint value, treatment).

load(“db.Rdata”).

data = db[,c(“record_id”,“endpoint”,“prot”)].

# Create a group variable (empty vector).

data$group = 0.

# Randomly sample one unit from the population.

rand = sample(data$record_id,1).

# Assign group 1 to this unit.

data$group[data$record_id =  = rand] = 1.

# Group 1 is assigned to all units that come from the same treatment as the selected patient.

data$group = ifelse(data$prot =  = data$prot[data$record_id =  = rand],1,0).

# Remove the treatment column from the dataset.

data = data[,-3].

# Compute the standard deviation of the endpoint (stratified by intervention group).

sd = aggregate(data$endpoint, by = list(data$group), FUN = sd).

# Print the results.

sd.

## Abbreviations

ANOVA: Analysis of variance
AR: As randomized
ARAT: Action research arm test
BCI: Brain-computer interface
CRF: Case report form
CTC: Clinical trial centre
EEG: Electroencephalography
EMA: European medicines agency
FDA: Food and drug administration
FSL: Fondazione Santa Lucia
FMA: Fugl-meyer assessment
ICH: International council for harmonisation
IPS: Internal pilot study
IRCCS: Istituti di Ricovero e Cura a Carattere Scientifico
ISS: Istituto Superiore di Sanità
ITT: Intention-to-treat
LASSO: Least absolute shrinkage and selection operator
MAS: Modified ashworth scale
MCID: Minimal clinically important difference
MEP: Motor evoked potential
MI: Motor imagery
MMT: Manual muscle test
MRI: Magnetic resonance imaging
NASA-TLX: NASA task load index
NIHSS: National institute of health stroke scale
NRS: Numeric rating scale
PP: Per-protocol
QCM: Questionnaire on current motivation
QUEST: Quebec user evaluation of satisfaction with assistive technology
RCT: Randomized controlled trial
SAP: Statistical analysis plan
SD: Standard deviation
SPIRIT: Standard protocol items: recommendations for interventional trials
SUS: System usability score
TAP: Test of attentional performance
TMS: Transcranial magnetic stimulation
TMT: Trail making test
TT: Token test
UE: User experience
UE-FMA: Fugl-meyer assessment for upper extremity
VAS: Visual analogue scale
